# 2-(Prop-2-en­yl)-1,2-benzisothia­zol-3(2*H*)-one 1,1-dioxide

**DOI:** 10.1107/S1600536809016328

**Published:** 2009-05-07

**Authors:** Muhammad Nadeem Arshad, Hafiz Mubashar-ur-Rehman, Muhammad Zia-ur-Rehman, Islam Ullah Khan, Muhammad Shafiq

**Affiliations:** a Department of Chemistry, Government College University, Lahore 54000, Pakistan; b Applied Chemistry Research Centre, PCSIR Laboratories Complex, Ferozpur Road, Lahore 54600, Pakistan

## Abstract

In the title compound, C_10_H_9_NO_3_S, the benzisothia­zole group is almost planar (with a maximum deviation of 1.61 Å). The crystal structure is stabilized by weak inter­molecular C—H⋯O hydrogen bonds, forming a chain of mol­ecules along *b*.

## Related literature

For the synthesis of benzothia­zine and benzisothia­zol deriv­atives, see: Zia-ur-Rehman, Anwar & Ahmad (2006 ▶); Zia-ur-Rehman, Anwar, Ahmad & Siddiqui (2006 ▶); Siddiqui *et al.* (2007 ▶) Zia-ur-Rehman *et al.* (2009 ▶). For the biological activity of benzisothia­zols, see: Kapui *et al.* (2003 ▶); Liang *et al.* (2006 ▶). For related structures, see: Siddiqui, Ahmad, Siddiqui *et al.* (2007*a* ▶,*b* ▶,*c* ▶).
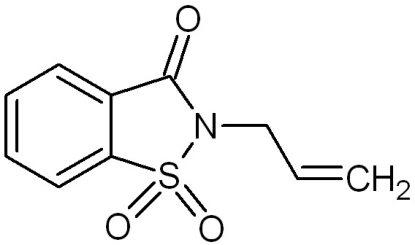


## Experimental

### 

#### Crystal data

C_10_H_9_NO_3_S
*M* *_r_* = 223.24
Triclinic, 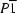

*a* = 7.2169 (8) Å
*b* = 7.8347 (7) Å
*c* = 10.3849 (12) Å
α = 105.530 (3)°
β = 91.586 (3)°
γ = 112.047 (3)°
*V* = 518.95 (10) Å^3^
*Z* = 2
Mo *K*α radiation
μ = 0.30 mm^−1^
*T* = 296 K
0.37 × 0.26 × 0.18 mm

#### Data collection

Bruker APEXII CCD area-detector diffractometer
Absorption correction: none
5460 measured reflections
2342 independent reflections
1728 reflections with *I* > 2σ(*I*)
*R*_int_ = 0.022

#### Refinement

*R*[*F* ^2^ > 2σ(*F* ^2^)] = 0.041
*wR*(*F* ^2^) = 0.118
*S* = 1.06
2342 reflections
136 parameters
H-atom parameters constrained
Δρ_max_ = 0.26 e Å^−3^
Δρ_min_ = −0.26 e Å^−3^

### 

Data collection: *APEX2* (Bruker, 2007 ▶); cell refinement: *SAINT* (Bruker, 2007 ▶); data reduction: *SAINT*; program(s) used to solve structure: *SHELXS97* (Sheldrick, 2008 ▶); program(s) used to refine structure: *SHELXL97* (Sheldrick, 2008 ▶); molecular graphics: *PLATON* (Spek, 2009 ▶) and *Mercury* (Macrae *et al.*, 2006 ▶); software used to prepare material for publication: *PLATON*.

## Supplementary Material

Crystal structure: contains datablocks I, global. DOI: 10.1107/S1600536809016328/bt2942sup1.cif

Structure factors: contains datablocks I. DOI: 10.1107/S1600536809016328/bt2942Isup2.hkl

Additional supplementary materials:  crystallographic information; 3D view; checkCIF report

## Figures and Tables

**Table 1:** Hydrogen-bond geometry (Å, °)

*D*—H⋯*A*	*D*—H	H⋯*A*	*D*⋯*A*	*D*—H⋯*A*
C6—H6⋯O1^i^	0.93	2.36	3.216 (3)	153

Symmetry code: (i) 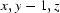.

**Fig. 1.**
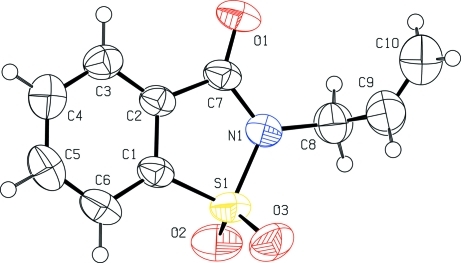
The molecular structure of the title compound with displacement ellipsoids at the 50% probability level.

**Fig. 2.**
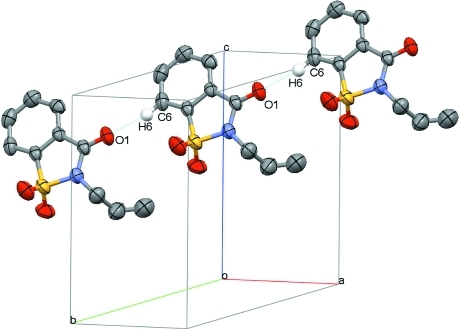
Perspective view of the crystal packing showing hydrogen-bonded interactions (dashed lines). H atoms not involved in hydrogen bonding have been omitted for clarity.

## supplementary crystallographic information

### Comment

Besides being used as a sweetener, saccharin and its various derivatives are well known for their different type of biological activities *e.g.*, it has been identified as an important molecular component in various classes of 5-HTla antagonists, analgesics and human mast cell tryptase inhibitors (Kapui *et al.*, 2003; Liang *et al.*, 2006). *N*-alkyl derivatives of saccharin have been successfully transformed to non-steroidal anti-inflammatory drugs *e.g.*, piroxicam and meloxicam.

As part of a research program synthesizing various bioactive benzothiazines (Zia-ur-Rehman *et al.*, 2009; Siddiqui *et al.*, 2007), we have in addition, worked on the synthesis of benzisothiazole derivatives. We herein report the crystal structure of the title compound (Scheme and figure 1). The benzisothiazole moiety is exactly planar. The molecular dimensions are in accord with the corresponding dimensions reported in similar structures (Siddiqui, Ahmad, Siddiqui *et al.*, 2007*a*; Siddiqui, Ahmad, Siddiqui *et al.*, 2007*b*; Siddiqui, Ahmad, Siddiqui *et al.*, 2007*c*). Each molecule is linked to its adjacent one through C—H···O contacts forming a chain of molecules along *b* (Figure 2).

### Experimental

A mixture of 2,3-dihydro-1,2-benzisothiazol-3-one-1,1-dioxide (1.83 g, 10.0 mmoles), dimethyl formamide (5.0 ml) and allyl bromide (1.20 g, 10.0 mmoles) was stirred for a period of one hour at 90°C. Contents were cooled to room temperature; poured over crushed ice to get white coloured precipitates which were filtered, washed and dried. Crystallization of the white precipitate   in methanol  afforded suitable crystals for X-ray studies.

### Refinement

H atoms  were placed in geometric positions (C—H distance = 0.93 to 0.96 Å) using a riding model with *U*_iso_(H) = 1.2 *U*_eq_(C).

### Crystal data

**Table:**

C_10_H_9_NO_3_S	*Z* = 2
*M**_r_* = 223.24	*F*(000) = 232
Triclinic, *P*1	*D*_x_ = 1.429 Mg m^−^^3^
Hall symbol: -P 1	Mo *K*α radiation, λ = 0.71073 Å
*a* = 7.2169 (8) Å	Cell parameters from 2362 reflections
*b* = 7.8347 (7) Å	θ = 3.1–27.3°
*c* = 10.3849 (12) Å	µ = 0.30 mm^−^^1^
α = 105.530 (3)°	*T* = 296 K
β = 91.586 (3)°	Needles, colourless
γ = 112.047 (3)°	0.37 × 0.26 × 0.18 mm
*V* = 518.95 (10) Å^3^	

### Data collection

**Table:**

Bruker APEXII CCD area-detector diffractometer	1728 reflections with *I* > 2σ(*I*)
Radiation source: fine-focus sealed tube	*R*_int_ = 0.022
graphite	θ_max_ = 27.5°, θ_min_ = 2.9°
φ and ω scans	*h* = −9→9
5460 measured reflections	*k* = −10→6
2342 independent reflections	*l* = −11→13

### Refinement

**Table:**

Refinement on *F*^2^	Primary atom site location: structure-invariant direct methods
Least-squares matrix: full	Secondary atom site location: difference Fourier map
*R*[*F*^2^ > 2σ(*F*^2^)] = 0.041	Hydrogen site location: inferred from neighbouring sites
*wR*(*F*^2^) = 0.118	H-atom parameters constrained
*S* = 1.06	*w* = 1/[σ^2^(*F*_o_^2^) + (0.0542*P*)^2^ + 0.1101*P*] where *P* = (*F*_o_^2^ + 2*F*_c_^2^)/3
2342 reflections	(Δ/σ)_max_ < 0.001
136 parameters	Δρ_max_ = 0.26 e Å^−^^3^
0 restraints	Δρ_min_ = −0.26 e Å^−^^3^

### Special details

**Table:**

Geometry. All e.s.d.'s (except the e.s.d. in the dihedral angle between two l.s. planes) are estimated using the full covariance matrix. The cell e.s.d.'s are taken into account individually in the estimation of e.s.d.'s in distances, angles and torsion angles; correlations between e.s.d.'s in cell parameters are only used when they are defined by crystal symmetry. An approximate (isotropic) treatment of cell e.s.d.'s is used for estimating e.s.d.'s involving l.s. planes.
Refinement. Refinement of *F*^2^ against ALL reflections. The weighted *R*-factor *wR* and goodness of fit *S* are based on *F*^2^, conventional *R*-factors *R* are based on *F*, with *F* set to zero for negative *F*^2^. The threshold expression of *F*^2^ > σ(*F*^2^) is used only for calculating *R*-factors(gt) *etc*. and is not relevant to the choice of reflections for refinement. *R*-factors based on *F*^2^ are statistically about twice as large as those based on *F*, and *R*- factors based on ALL data will be even larger.

### Fractional atomic coordinates and isotropic or equivalent isotropic displacement parameters (Å^2^)

**Table:**

	*x*	*y*	*z*	*U*_iso_*/*U*_eq_	
S1	0.39784 (8)	0.35030 (7)	0.26041 (5)	0.0536 (2)	
O1	0.2640 (2)	0.6659 (2)	0.09710 (17)	0.0646 (4)	
O2	0.6073 (2)	0.3953 (2)	0.29080 (18)	0.0759 (5)	
O3	0.2677 (3)	0.2648 (2)	0.34679 (16)	0.0726 (5)	
N1	0.3652 (3)	0.5452 (2)	0.25013 (17)	0.0515 (4)	
C1	0.3079 (3)	0.2289 (3)	0.0897 (2)	0.0450 (4)	
C2	0.2602 (3)	0.3453 (3)	0.0270 (2)	0.0431 (4)	
C3	0.1889 (3)	0.2789 (3)	−0.1090 (2)	0.0533 (5)	
H3	0.1557	0.3555	−0.1521	0.064*	
C4	0.1684 (3)	0.0951 (3)	−0.1791 (2)	0.0635 (6)	
H4	0.1223	0.0479	−0.2713	0.076*	
C5	0.2147 (3)	−0.0205 (3)	−0.1155 (3)	0.0645 (6)	
H5	0.1983	−0.1442	−0.1656	0.077*	
C6	0.2844 (3)	0.0433 (3)	0.0204 (2)	0.0572 (6)	
H6	0.3145	−0.0347	0.0636	0.069*	
C7	0.2931 (3)	0.5357 (3)	0.1224 (2)	0.0463 (5)	
C8	0.4052 (4)	0.7114 (3)	0.3697 (2)	0.0642 (6)	
H8A	0.4591	0.8293	0.3443	0.077*	
H8B	0.5060	0.7163	0.4359	0.077*	
C9	0.2176 (5)	0.6993 (4)	0.4312 (3)	0.0823 (8)	
H9	0.1606	0.5989	0.4683	0.099*	
C10	0.1299 (5)	0.8116 (5)	0.4373 (3)	0.0945 (9)	
H10A	0.1814	0.9141	0.4015	0.113*	
H10B	0.0134	0.7926	0.4777	0.113*	

### Atomic displacement parameters (Å^2^)

**Table:**

	*U*^11^	*U*^22^	*U*^33^	*U*^12^	*U*^13^	*U*^23^
S1	0.0584 (3)	0.0538 (3)	0.0598 (4)	0.0253 (2)	0.0088 (2)	0.0306 (3)
O1	0.0772 (10)	0.0506 (8)	0.0818 (11)	0.0342 (7)	0.0108 (8)	0.0319 (8)
O2	0.0616 (10)	0.0850 (11)	0.0897 (12)	0.0328 (8)	−0.0054 (9)	0.0362 (10)
O3	0.0904 (12)	0.0726 (10)	0.0647 (10)	0.0278 (9)	0.0214 (9)	0.0418 (9)
N1	0.0612 (10)	0.0455 (9)	0.0537 (10)	0.0248 (8)	0.0095 (8)	0.0187 (8)
C1	0.0420 (10)	0.0435 (9)	0.0588 (12)	0.0201 (8)	0.0149 (9)	0.0249 (9)
C2	0.0391 (10)	0.0428 (9)	0.0554 (11)	0.0178 (8)	0.0139 (8)	0.0243 (9)
C3	0.0468 (11)	0.0607 (12)	0.0572 (13)	0.0207 (9)	0.0085 (9)	0.0262 (10)
C4	0.0540 (13)	0.0652 (14)	0.0616 (14)	0.0180 (11)	0.0098 (11)	0.0117 (11)
C5	0.0568 (13)	0.0470 (12)	0.0819 (17)	0.0195 (10)	0.0182 (12)	0.0080 (11)
C6	0.0514 (12)	0.0468 (11)	0.0845 (17)	0.0249 (9)	0.0194 (11)	0.0285 (11)
C7	0.0452 (10)	0.0429 (10)	0.0600 (12)	0.0198 (8)	0.0134 (9)	0.0259 (9)
C8	0.0702 (15)	0.0559 (12)	0.0604 (14)	0.0234 (11)	0.0031 (11)	0.0108 (11)
C9	0.111 (2)	0.0722 (16)	0.0749 (18)	0.0451 (16)	0.0323 (16)	0.0258 (14)
C10	0.109 (2)	0.098 (2)	0.0787 (19)	0.0495 (19)	0.0176 (17)	0.0177 (17)

### Geometric parameters (Å, °)

**Table:**

S1—O2	1.4220 (16)	C4—C5	1.379 (3)
S1—O3	1.4253 (15)	C4—H4	0.9300
S1—N1	1.6596 (16)	C5—C6	1.374 (3)
S1—C1	1.743 (2)	C5—H5	0.9300
O1—C7	1.206 (2)	C6—H6	0.9300
N1—C7	1.385 (3)	C8—C9	1.495 (3)
N1—C8	1.467 (3)	C8—H8A	0.9700
C1—C6	1.382 (3)	C8—H8B	0.9700
C1—C2	1.384 (2)	C9—C10	1.253 (4)
C2—C3	1.376 (3)	C9—H9	0.9300
C2—C7	1.481 (3)	C10—H10A	0.9300
C3—C4	1.378 (3)	C10—H10B	0.9300
C3—H3	0.9300		
			
O2—S1—O3	117.16 (10)	C6—C5—C4	121.4 (2)
O2—S1—N1	109.80 (9)	C6—C5—H5	119.3
O3—S1—N1	109.80 (9)	C4—C5—H5	119.3
O2—S1—C1	111.86 (10)	C5—C6—C1	116.9 (2)
O3—S1—C1	112.76 (9)	C5—C6—H6	121.5
N1—S1—C1	92.73 (8)	C1—C6—H6	121.5
C7—N1—C8	123.33 (17)	O1—C7—N1	123.46 (19)
C7—N1—S1	115.04 (13)	O1—C7—C2	127.23 (19)
C8—N1—S1	121.60 (14)	N1—C7—C2	109.31 (15)
C6—C1—C2	122.1 (2)	N1—C8—C9	111.41 (19)
C6—C1—S1	127.33 (16)	N1—C8—H8A	109.3
C2—C1—S1	110.60 (14)	C9—C8—H8A	109.3
C3—C2—C1	120.34 (18)	N1—C8—H8B	109.3
C3—C2—C7	127.38 (17)	C9—C8—H8B	109.3
C1—C2—C7	112.27 (17)	H8A—C8—H8B	108.0
C2—C3—C4	117.8 (2)	C10—C9—C8	126.1 (3)
C2—C3—H3	121.1	C10—C9—H9	116.9
C4—C3—H3	121.1	C8—C9—H9	116.9
C3—C4—C5	121.5 (2)	C9—C10—H10A	120.0
C3—C4—H4	119.3	C9—C10—H10B	120.0
C5—C4—H4	119.3	H10A—C10—H10B	120.0
			
O2—S1—N1—C7	112.66 (16)	C7—C2—C3—C4	−179.51 (17)
O3—S1—N1—C7	−117.16 (15)	C2—C3—C4—C5	0.9 (3)
C1—S1—N1—C7	−1.76 (15)	C3—C4—C5—C6	−0.4 (3)
O2—S1—N1—C8	−69.00 (18)	C4—C5—C6—C1	−0.6 (3)
O3—S1—N1—C8	61.18 (18)	C2—C1—C6—C5	1.2 (3)
C1—S1—N1—C8	176.58 (16)	S1—C1—C6—C5	−178.53 (15)
O2—S1—C1—C6	69.07 (19)	C8—N1—C7—O1	2.9 (3)
O3—S1—C1—C6	−65.5 (2)	S1—N1—C7—O1	−178.81 (15)
N1—S1—C1—C6	−178.31 (17)	C8—N1—C7—C2	−177.24 (16)
O2—S1—C1—C2	−110.65 (14)	S1—N1—C7—C2	1.1 (2)
O3—S1—C1—C2	114.77 (14)	C3—C2—C7—O1	−0.5 (3)
N1—S1—C1—C2	1.96 (14)	C1—C2—C7—O1	−179.66 (19)
C6—C1—C2—C3	−0.7 (3)	C3—C2—C7—N1	179.65 (18)
S1—C1—C2—C3	179.07 (14)	C1—C2—C7—N1	0.5 (2)
C6—C1—C2—C7	178.58 (16)	C7—N1—C8—C9	84.2 (3)
S1—C1—C2—C7	−1.68 (19)	S1—N1—C8—C9	−94.0 (2)
C1—C2—C3—C4	−0.4 (3)	N1—C8—C9—C10	−114.2 (3)

### Hydrogen-bond geometry (Å, °)

**Table:**

*D*—H···*A*	*D*—H	H···*A*	*D*···*A*	*D*—H···*A*
C6—H6···O1^i^	0.93	2.36	3.216 (3)	153

Symmetry codes: (i) *x*, *y*−1, *z*.
